# Initial Commitment to Pre-Exposure Prophylaxis and Circumcision for HIV Prevention amongst Indian Truck Drivers

**DOI:** 10.1371/journal.pone.0011922

**Published:** 2010-07-30

**Authors:** John A. Schneider, Rakhi Dandona, Shravani Pasupneti, Vemu Lakshmi, Chuanhong Liao, Vijay Yeldandi, Kenneth H. Mayer

**Affiliations:** 1 Department of Medicine, University of Chicago, Chicago, Illinois, United States of America; 2 Department of Health Studies, University of Chicago, Chicago, Illinois, United States of America; 3 Public Health Foundation of India, New Delhi, India; 4 Pritzker School of Medicine, University of Chicago, Chicago, Illinois, United States of America; 5 Department of Microbiology, Nizam's Institute of Medical Sciences, Hyderabad, India; 6 SHARE-India, Hyderabad, India; 7 Department of Medicine, Brown University, Providence, Rhode Island, United States of America; 8 Department of Community Health, Brown University, Providence, Rhode Island, United States of America; CIET, Canada

## Abstract

Studies of HIV prevention interventions such as pre-exposure prophylaxis (PREP) and circumcision in India are limited. The present study sought to investigate Indian truck-drivers initial commitment to PREP and circumcision utilizing the AIDS Risk Reduction Model. Ninety truck-drivers completed an in-depth qualitative interview and provided a blood sample for HIV and HSV-2 testing. Truck-drivers exhibited low levels of initial commitment towards PREP and even lower for circumcision. However, potential leverage points for increasing commitment were realized in fear of infecting family rather than self, self-perceptions of risk, and for PREP focusing on cultural beliefs towards medication and physicians. Cost was a major barrier to both HIV prevention interventions. Despite these barriers, our findings suggest that the ARRM may be useful in identifying several leverage points that may be used by peers, health care providers and public health field workers to enhance initial commitment to novel HIV prevention interventions in India.

## Introduction

South/Southeast Asia is the second-most HIV affected region in the world, and India continues to have the highest numbers of HIV infected in Asia [1]. Evidence from the most recent National Family Health Survey [2], demonstrates that the state of Andhra Pradesh (AP) has the highest prevalence of HIV infection due to sexual transmission in India [3]. Overall estimates of HIV infection rates in India reflect an epidemic in high-risk populations and have created a shift in resources to focus on prevention efforts as evidenced by the recent National AIDS Control Program-3 (NACP-3) implementation plan for 2007–2012 which allocates 67.2% of the entire budget to HIV prevention efforts [4].

Early in the HIV pandemic, truck drivers along the trans-African highways and in Asia were increasingly recognized as a high risk bridge population for HIV infection [5], [6], [7]. With 3 million trucks, often with both a driver and younger male helper or cleaner, on the roads in India [8], there are surprisingly few research studies examining methods for preventing HIV in this population [9] [10].

Current HIV prevention programs in India have been heavily condom-centric [11]. While condoms are one of the most efficacious of HIV Prevention Interventions currently available, they are often not the most effective due to poor adherence [12]. Condom use amongst men remains at 5% [2] with condom promotion efforts in India having been limited by several historical, cultural and health belief factors including British population control promotion [13]. Additionally, for truck-drivers who already are forced to endure high engine temperatures while on the road, the belief that condom use may block the flow of semen and cause an unnatural rise in body heat for men, may be viewed as detrimental to health [14].

With the expectation that it may take years to demonstrate the efficacy of a highly effective preventive vaccine, [15] increased attention has been focused on other biological interventions [16]. Animal and human data support the use of post-exposure antiretroviral therapy to decrease HIV incidence [17], [18], and efficacy trials are currently underway to evaluate PREP (taking HIV medication to prevent HIV infection). Circumcision has been shown to decrease the risk of HIV acquisition in men by 50–60% in three randomized controlled trials from Africa [19], [20], [21]. Male centered HIV prevention interventions such as circumcision or those related to pill ingestion such as PREP may be potential candidates for mobile populations such as truck-drivers that are at increased risk of HIV acquisition.

However, it is anticipated that these new HIV prevention strategies and technologies will need to be carefully assessed in specific populations because 1) these advances are unlikely to provide full protection against HIV [21]; 2) the possibility of risk compensation (adjustment in behavior due to perceived changes in risk) or behavioral disinhibition (failure to inhibit behavior when aversive consequences are likely) could mitigate the benefits [22]; 3) strategies using medication will require ongoing adherence [22]; 4) they may offer little or no protection against other sexually transmitted infections [16]; 5) they likely will provide either variable protection against unintended pregnancies or limit the ability to become pregnant and 6) each intervention has to be introduced carefully into local environment paying special attention to local cultural beliefs, customs and community opinions [23]. Warnings by the former UNAIDS director that Hindus in India may be more likely to become HIV infected because of uncircumcised status created significant controversy and may have resulted in increasing negative attitudes towards circumcision [23], [24]. Additionally, “social expectations” of prevention interventions like PREP have recently gained attention and require early planning and assessment even prior to our understanding of their potential for effectiveness [25].

Studies of initial commitment to and effectiveness of PREP and circumcision in India are limited. Current studies of PREP are taking place outside of India (www.avac.org) and other than one modeling study which demonstrated the potential for 2700 averted HIV cases in Southern India [26], there has been no examination of initial commitment to PREP nor have efficacy studies yet been planned. Although one study of mothers found some willingness to circumcise their infant sons [27], circumcision has not been examined in high-risk adult populations.

In this study we examine initial commitment towards PREP and circumcision, amongst truck-drivers in India with the hypotheses that initial commitment is based largely upon self-perception of risk and local cultural attitudes. PREP and circumcision are two biomedical HIV prevention interventions respectively – one which has generated a significant amount of interest but is yet to be proven efficacious and the other which has been proven to be efficacious, but has been met with some controversy in specific settings. We utilize the first two stages of the ARRM as our theoretical framework and a mixed methods approach to identify emerging themes, peer acceptability and potential leverage points for future intervention.

### Conceptual Framework

In this study, the AIDS risk reduction model (ARRM) [28] is used to guide questions on labeling and initial commitment to circumcision and pre-exposure prophylaxis (PREP) for HIV prevention. The ARRM is grounded in the theory of planned behavior [29], social cognitive theory [30], the health belief model [31] and the diffusion of innovation model. The ARRM is represented as a three stage process that focuses on risk assessment, factors that influence the decision to reduce risk, and the ability to change and maintain new behavior. A complete review of the ARRM and its implications was first described nearly 20 years ago [28].

**Figure 1:**
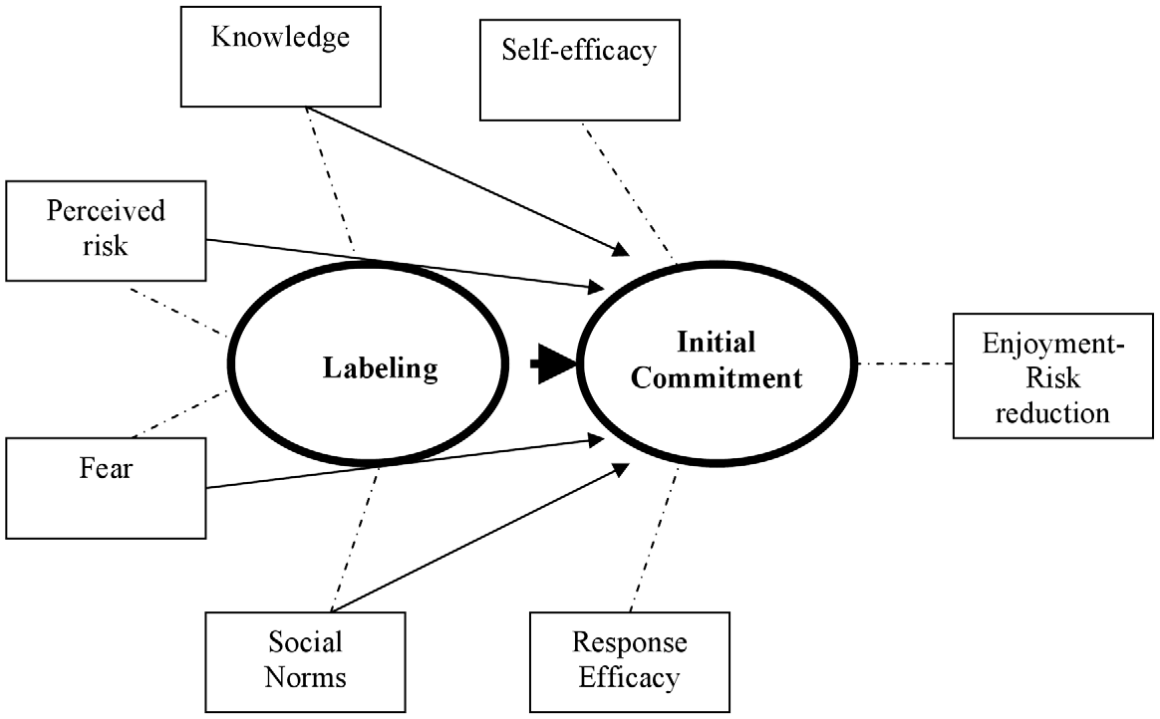
Conceptual framework outlining development of commitment for the use of two novel HIV prevention interventions, PREP and circumcision, through the first two stages of the AIDS Risk Reduction Model. Circles represent stages and squares represent domains of stages. Arrows represent domains/stages that affect a future stage. Dashed lines represent domains linked to stages. Labeling includes general factors that may exist in addition to circumcision and PREP. Implicit to this model is the effect of culture and geographical context on stages and domains.

The ARRM has led to useful empirically supported insights regarding leverage points for risk behavior change and has identified correlates of HIV risk behavior that should be considered in designing interventions [32]. Progression through ARRM stages is dependent on the influence of several psychosocial factors as depicted in Figure 1. In stage 1, “labeling”, perception of risk is mediated by the following constructs: an individual understands how HIV is transmitted sexually, believes they are susceptible to the disease, believes that HIV is undesirable and their social networks reinforce these perceptions. In stage 2, “developing commitment”, factors affecting the decision-making process are based upon the following influences: belief in the effectiveness of behaviors in reducing the risk of disease, reflections of how behaviors affect sexual pleasure, ability to affect the risk behavior and social norms that support or inhibit commitment to behavior change. Ability to affect the behavior is posited as a critical predictor of intention to change at stage 2, as well as actual behavior change at stage 3.

Studies that utilize the ARRM theoretical framework have mostly assessed developing commitment to changes in sexual risk taking behavior, adoption of condom use, and utilization of clean needles [33] [34]. Severy et al. have provided a framework for utilization of the ARRM for assessing the acceptability and maintenance of use of novel candidate microbicides, however, this framework has not been formally assessed [35]. The ARRM theoretical framework has also not been applied to other candidate HIV prevention interventions such as the ones examined in this study, nor has it been tested in Asia. In this study using a mixed methods approach, we applied the ARRM framework to assess proximate factors that might affect future uptake of circumcision or PREP.

## Methods

### Ethics Statement

Procedures and protocols were approved by co-investigators' institutional review boards (including the first author) in India (SHARE-India) and the United States (The Miriam Hospital and the University of Chicago). All study participants provided written informed consent.

### Participants and setting

This study was conducted at a large transport depot center and associated 4 parking lots on the outskirts of Hyderabad, the capital of Andhra Pradesh (AP) India between 2007 and 2009, to assess initial commitment of truck drivers to two candidate HIV prevention interventions. Truck drivers were selected, as AP has the highest rates of HIV infection due to heterosexual transmission in the country, and truck-drivers have among the highest male heterosexual prevalence [3]. Truck-drivers between the ages of 18–55, who were fluent Hindi or Telugu speakers, reported they were either HIV negative or had never been tested before were eligible for participation. Truck-drivers were individually approached in one of four parking lots over four day cycles at different times of day [36]. Participants were approached from a non-randomly generated sample, as was the practice of the concurrently run *Surakshitham Project*, a government funded HIV prevention program for truck-drivers [37]. One of two trained research assistants asked candidate truck-drivers if they were interested in participating in a behavioral risk survey followed by HIV-1/2 and Herpes Simplex Virus Type 2 (HSV-2) testing. Testing of HIV – 1/2 and HSV-2, in this group of men who had not been tested previously, was conducted to complement self-reported sex behavior and provide another measurement to determine an objective level of previous risk taking behavior.

### Data collection procedures

The interviews were administered in person in a sound-proof room by one of three trained research assistants all with 6 years experience working with this population. Each research assistant had previous experience in qualitative interviews and was further trained by reading a training manual and watching a videotape of a model interview. Basic sociodemographics were collected prior to the interview. The research assistants were given a script to follow, which contained semi-structured interview questions (**Figure S1**). They were encouraged to probe participants further when responses were vague or unclear. The interview questions inquired about truck-driver and peer HIV knowledge, perception of risk, sexual experiences, social norms, self-efficacy, knowledge, attitudes, beliefs and initial commitment towards PREP and circumcision. Circumcision and PREP were chosen for this population because they were novel HIV prevention interventions under investigation at the time of the study, and they presented very different implementation challenges. Estimates of circumcision efficacy (50–60%) were based upon results from three randomized trials [19], [20], [21] and estimates of PREP efficacy (50%) was based upon modeling studies [26], [38], with adjustments made for potential non-adherence and comparability, resulting in using a 50% estimate of efficacy for each intervention. Pilot cognitive testing of survey items demonstrated that 50% protection was easier for study participants to comprehend than 60% usually ascribed to the protective effect of circumcision.

Blood samples were collected by a dry blood spot fingerstick technique and tested for HSV-2 and HIV 1/2 antibodies on all participants using standard methods for detecting HSV-2 (HerpeSelect 2 ELISA IgG, Focus Diagnostics, CA) and HIV 1/2 (Vironostika HIV Uniform II AG/AB, bioMérieux, France) (Murex HIV; Murex Biotech) (HIV Tridot; J. Mitra, New Delhi, India) following pre-test counseling. Following written informed consent, the interviews and dry blood spot collection took approximately ninety minutes to complete, and participants were provided a token gift (bag of rice or blanket) worth 4 USD (approximately 200 Indian Rupees) for their participation. Each interview was digitally recorded and professionally transcribed verbatim into Telugu or Hindi. Transcripts were then translated into English by a local doctoral level anthropology or english consultant translator. If participants were interested in results to testing, they were provided post-test counseling by trained counselors, with referrals to a linked government hospital for further management.

### Qualitative measures and analysis

Interview transcripts were analyzed by employing an iterative process of qualitative textual analysis. Open coding [39] was first utilized to code the initial interviews by identifying and labeling discrete units of text to generate themes related to attitudes towards PREP and circumcision. Following this process the interviews were all coded using an analytic induction technique [40] that referred to one or more domains relevant to the ARRM including initial commitment towards circumcision and PREP. Three members of the study team met to develop a consensus about the content of and appropriate names for the different concepts and to formulate a working codebook of primary ARRM domains, and emerging themes. ARRM domains included those in **Figure 1**, and were coded for examples both positive and negative for each domain. After the first few iterations of this process, and when the codebook developed some stability (9% of interviews), we utilized qualitative software (NVivo qualitative data analysis software; QSR International Pty Ltd. Version 8, 2008) to recode all prior and subsequent interviews according to the codebook formulations. Where new ideas and themes emerged, we made addenda to the codebook. At various points throughout the study, an inductive approach was employed to identify emergent themes and to identify relationships and patterns between the themes. Following the principle of constant comparison [41] each new transcript was considered in relation to the prior ones to ensure that the codebook and our evolving interpretations remained faithful to the data. All 90 interviews were coded using this scheme by S.P. A random selection of 25 participants was subjected to analysis of inter-coder agreement on labeling classification using k coefficient which indicated “substantial” agreement[42]: k = 0.91. Discrepancies were resolved by reviews of the transcripts and reflective discussions.

### Quantitative measures

The following sociodemographic and behavioral characteristic variables were collected and categorized according to previous work: age, education, income, religion, circumcision status, marital status, contact with female sex workers, history of a sexually transmitted disease [9], [43]. Sociodemographic variables were dichotomized for bivariate analyses. After assessing baseline knowledge attitudes and beliefs towards PREP and circumcision, the interviewers delivered set paragraphs describing circumcision and PREP in the context of HIV prevention with a “teach back” approach [44] until participant comprehension was achieved. Caution was followed to ensure that participants understood that there was uncertainty for the potential effectiveness of these two interventions in India, potential for side effects, and that condoms would still be required. Circumcision was described as a minor surgical procedure, and PREP as a medication that has to be taken every day, and both with 50% efficacy. Participants were then asked about their initial commitment to PREP or circumcision using the following question: Are you ready to get circumcised (take PREP) to reduce your risk for getting HIV?

A willingness to pay application was utilized to determine a second measure of the acceptability of circumcision and PREP between individuals and in those who were not interested in the interventions for their personal use. Willingness to pay is the maximum amount a person will be willing to pay for a good - in this case PREP and circumcision. The reason for using this application was to determine relative subjective values of the prevention interventions amongst study participants and not upon absolute values and comparisons of the two prevention interventions to one another other. Participant willingness to pay for PREP was based upon the response to the following question: How much would you be willing to pay for PREP each month from now on to prevent HIV infection? Participant willingness to pay for circumcision was based upon the response to the following question: How much would you be willing to pay for a one time circumcision to prevent HIV infection? To determine social acceptability and norms of the interventions in those who were not interested in circumcision and/or PREP for personal use, study participants who were not willing to use the prevention interventions based upon previous initial commitment questioning were asked to indicate what a peer who was ready to use these interventions might pay. Cost response categories for PREP and circumcision were first modeled around the mean value of market prices for both entities from three local health care institutions at the time of the study. Focus group discussions demonstrated that these costs were largely out of reach for most truck-drivers and thus categories were decreased by a factor of 30 for PREP and 3 for circumcision in order to maximize likelihood of variability in responses. Final categories were 0–25, 26–50, 51–100, 101–150, 151–200, >200 for PREP and 0–500, 501–1000, 1001–1500, 1501–2000, 2001–2500 and >2500 (40 rupees = $1) for circumcision. After choosing a category, participants were asked to provide a specific rupee amount within the category. Models for cost of lifetime PREP were adjusted for present discounted value (PDV) (i), and adjusted by an estimate of future years of sexual activity and PREP coverage [26] (ii):

- Discount factor  = 1/(1+r); monthly discount rate (r)  = 0.004
- Lifetime PREP cost  =  
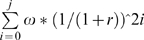
;

*ω* = cost/month, j = (50-age)*6

The level of lifetime sexual activity in this population was based on local data from demographic studies [45] and ongoing analyses from our group [43]. Estimates of lifetime PREP adherence at 50% were conservative and based upon previous PREP modeling work which elicited a coverage rate of 50% as a conservative estimate in South India [26].

### Integration of methods and analysis

The analytic framework used in this study focused on complementarity [46]. Overlapping but different facets of initial commitment were measured in order to yield an enriched, elaborated understanding of that phenomenon and potential leverage points for future intervention. Qualitative analyses focused on the domains within the ARRM that contribute to one's initial commitment. Next complementary bivariate analyses were performed comparing sociodemographic and risk factor variables to initial commitment to both HIV prevention interventions. Finally, the willingness to pay application was analyzed to determine acceptability of the two prevention interventions amongst those not interested in using them personally. Initial commitment was elicited by directly asking study participants about their potential commitment. A modified Poisson regression approach was utilized to determine relative risks [47] and the Wilcoxon rank-sum test was utilized in bivariate analyses of non-normal data. A p-value of 0.10 was reported to suggest borderline significance and a final p-value of 0.05 was used to determine statistical significance. All quantitative data analyses were performed using Stata version 9.1 (College Station, Texas).

## Results

### Sample

A convenience sample of ninety truck-drivers from 204 approached (44.1%) were enrolled in the study. Leading reasons for refusal included lack of time due to loading/unloading truck, repairing/cleaning truck, cooking/rest time, and concern over confidentiality. Of participants, Telugu was the primary language of 61.1% of the drivers and Hindi for the remaining. HIV-1/2 and HSV-2 seroprevalence of participants was 2.2% and 5.6% respectively, with HIV prevalence similar to another study in this setting (2.1%) [9]. The demographics of the participants are detailed in **Table 1**.

**Table 1:** Sociodemographic characteristics of truck-drivers in sample (n = 90).

Characteristic	N (Percent of Total)
Age (years) (range 19–52)	
<25	20 (22.5)
25–29	29 (32.6)
30–34	13 (14.6)
35–40	12 (13.5)
>40	15 (16.9)
Marital status	
Married	79 (87.8)
Separated/Divorced/Widowed	2 (2.2)
Unmarried	9 (10.0)
Education	
None	15 (16.9)
Class 1–10	36 (40.5)
Class 11–12	33 (37.1)
> Class 12	5 (5.6)
Monthly income (Indian Rupees)	
<2999 (∼$65)	14 (15.7)
3000–4499 (∼$65–$95)	28 (31.5)
4500–5999 (∼$95–$125)	27 (30.3)
≥6000 (∼$125)	20 (22.5)
Religion	
Hindu	71 (78.9)
Muslim	17 (18.9)
Christian	2 (2.2)
Circumcision status	
Uncircumcised	73 (83.9)
Circumcised	14 (16.1)

### Sub-domains within Labeling and Initial Commitment

Emerging themes within sub-domains are presented below and follow stages within the ARRM.

### Labeling - Knowledge

Study participants were very aware (97.8%) of HIV and major modes of transmission and slightly less so for other sexually transmitted infections (83.3%). All participants aware of HIV described the increased risk for HIV infection as occurring in extramarital relationships that were designated as “outside” or “immoral” relationships. Erroneous information, such as “HIV is transmitted through mosquitoes”, was rare, and did not involve information that if acted upon would not affect HIV transmission or acquisition. Most knowledge was acquired through discussions with peers and others at the workplace (75.9%).

### Labeling - Perceived Risk

Most truck-drivers (77.9%) perceived HIV to be affecting their profession, and (51.3%) personally knew another driver who was infected. Moreover, the overwhelming majority of truck-drivers perceived themselves to be at low risk, largely because any risky behavior was in the past before marriage (88.9%). Drivers' sexual encounters were usually referred to as occurring in the *past “before my marriage I went to prostitutes many times”*, and ongoing encounters or unplanned encounters were often deemed less risky, because of a known partner *“she is from my village”* or *“nice girl”* or *“the one who I was in love with once”*.

Not having sex outside of marriage or with sex workers was the main indicator for the perception that one was at low risk for HIV acquisition and thus a lack of initial commitment for PREP or circumcision. *“It (PREP) is good for them (other drivers who visit sex workers), sir…but for the ones who'll never do such thing there is no meaning in taking these medicines…I go home…do it there…there is no meaning in taking….”* Understanding that the differential use of PREP may depend upon the type of sexual encounter was also evident. “*If I go see a prostitute, only then would I use the medicine. I do not have any interest in using it with my wife.*” Motivation of preventive behavior adoption was evident with perception of risk for HIV infection that a female sex worker might pose. There were no obvious themes invoked by participants that were consistent with behavioral disinhibition or risk compensation.

### Labeling - Fear

HIV/AIDS was the most reported health condition (46.2%) that was feared in response to the open ended question “What health conditions or diseases do you fear? Why?” Avoidance of risk behavior was largely in the context of fear of infecting family more than the undesirability of infecting self. *“I am not afraid, if it spoils my life. I am afraid of it because it not only spoils my life but also children's life…..and spoils my wife, daughters and sons health. It means it is very dangerous to whole family. Why I spoil the whole family because of this small thing.”* When there was concern for infecting self, it often reflected back on the family as in loss of income and inability to provide for dependants. *“Brother, if you were to get sick, then you would not be able to make a living. Nothing will be able to happen. You've got a wife and kids to look after…..You won't be able to eat yourself and you won't be able to feed them either…A man should be free of that.”*

### Labeling - Social Norms

Strong social normative forces were related to most choices about sexual decision making and preventive behaviors. Networks that reinforced behavior consisted of peers involved in the transport business and negative behaviors were often mentioned in relation to the occupation. In the context of occupation effects and being away from home, having sex outside the marriage was normative and routine, with 77.9% of drivers stating that HIV/AIDS was part of the transport profession. *“More than any other illness, a truck driver has a chance of contracting AIDS, because truck drivers solicit a lot of prostitution on the road…..I would say that approximately 75% of them do it…..So they keep on doing it. This is the only thing that they enjoy. That is why they will more often have AIDS.”*

Strong cultural forces were present amongst participants and reflected mostly within the context of commitment towards circumcision. The majority of explanations of circumcision were matter of fact, “*this is not in our custom, sir. We are Hindus*”, however, there were participants who felt more strongly than others: *“This is not followed in our Hindu religion. Even if you offer millions of rupees, we do not go for cutting off the foreskin…… even then (in case of foreskin obstruction) we do not get ourselves circumcised.”* Religious norms overwhelmed any other new framework for conceptualizing circumcision such as a new method for HIV prevention, as well as existing frameworks such as circumcision improves hygiene. Hindu men recognized that circumcision may be more hygienic and this was often a topic that was discussed in the context of initial commitment amongst all but one uncircumcised man who suggested that he would be interested in circumcision. However, for the majority of uncircumcised men, religious norms did overwhelm any potentially positive hygienic qualities of circumcision. *“If the skin was not cut…. in urgency we cannot clean inside of the penis… So possibility is there to spread filth and chance to get disease…..The knowledge about it is correct sir. But if my village people know this… their behaviour towards me will change…”* This notion of ability to be identified and categorized based upon circumcision status was portrayed within a larger communal context. *“This helps in distinguishing between Hindus and Muslims. Is this a Hindu or Muslim? So they will say, “Look on his penis. Is he circumcised or not?” So they open his pants and look. Yes, brother, he is circumcised. So bury him. If he is uncircumcised, then he is a Hindu. Burn him. I have seen it in a movie, sir.”*

Muslim men found the relationship between circumcision and HIV to not be surprising with reasons for circumcision being multi-factorial including health, tradition and a mechanism for gaining respect. *“It is good for one's health. And for our tradition as well… They take it off for devotedness. The elders do it as well. He has gotten older, he has gotten older, they say.”* In contrast, any cultural explanations for the potential use or non-use of PREP were not mentioned by study participants during the qualitative interviews.

### Initial Commitment - Self-efficacy

Lack of control over one's behavior and life trajectory was common amongst truck-drivers. The overwhelming majority (75.6%) described the occupation as a mediating factor which has negative effects on health with little ability for change. *“A man is unable to find employment or break into a line of business, so he is forced to drive. Now one does not eat, does not bathe, regularity continues to be a problem. Thus, he has problems with his health.”* Additionally, there was a general perception of a lack of ability to change negative sex behavior outcomes because of the line of work. *“Truck drivers have a lot of work sir. My house is on the road. I can stop at home going from this direction as well… and I can stop there coming from that direction as well. The rest of these drivers will go home once in six months. If they don't do bad (immoral/wrong) things on the road, then where will they go?”*

### Initial Commitment - Response Efficacy

Respondents largely believed that PREP would work. “*In case nothing wrong happens to us after using this tablet then we feel that this tablet is good. Next time if an occasion arises, we then think of using this tablet. This is what we feel then.*” Additionally, recommendations of the efficacy of PREP from a physician were important. “*after that doctor says that – you need this tablet I will write the name of the tablet go to the medical store and take it.*” These sort of recommendations from physicians seemed to be more powerful than commonly used public service recommendations for current HIV prevention interventions such as condoms. Of note, there were no cases where circumcision was mentioned in this way and may reflect the connection between medications and physicians and less of a connection between circumcision and physicians. However, the concept of 50% efficacy was seen as a weakness of the product. “*50% only*” and *“..it tries to prevent the HIV, but it is not completely prevents the disease…….what is the use to take the medicine…..?”* Thus the efficacy modeled in this study at 50%, may engender poor commitment towards PREP.

Amongst uncircumcised participants, explanatory models of why circumcision was effective were ascribed to biological models with both lack of space for the virus to stay and healing explaining the protective response: *“If we think logically also, when the foreskin folds backwards while doing sex, in the case of an uncircumcised person, the virus may get into the folds and remain there.”* and *“healing (after STD) will be faster”.*

### Initial Commitment - Enjoyment Risk Reduction

Concerns about side effects were raised more frequently with PREP. “*Like… If I were to take a pill right now…After I take the pill, suppose that I didn't visit with any woman (for sex) …What kind of problems can the pill cause? ……Can there be any problem in the body afterward?*” Additionally, concerns about sexual performance after taking the pill were mentioned. “*In eating this tablet… before having sex, I will take the tablet. In this, there is no obstacle in sex?*” Because circumcision was frequently practiced in a large minority group and awareness was already high, side effects of surgery were not mentioned as a potential negative aspect or loss of enjoyment.

Hygiene benefits were the most recognized theme that would increase overall enjoyment after circumcision (33%) amongst circumcised and uncircumcised men. There could be improvement in sex because of a hygienic state. One uncircumcised man said, “*Sex would still be enjoyable. Genitals stays clean. After having a circumcision, it stays clean. Otherwise filth spreads in it.*” Also, except for one uncircumcised participant, there were generally no fears that sex would not remain enjoyable and there would not be pain with intercourse after circumcision. Circumcised men unilaterally failed to mention any perception of loss of enjoyment, and amongst uncircumcised men they were either not worried about loss of enjoyment or believed that it would not affect pleasure of intercourse and may even keep the penis clean.

### Determinants of initial commitment to PREP and circumcision

Overall 32.9% of study participants described initial commitment towards PREP and 11.1% of uncircumcised participants reported initial commitment towards circumcision. The relationship between commitment towards PREP and commitment towards circumcision did not reach statistical significance (OR 2.7; p = 0.15). The relationship between sociodemographic characteristics and risk correlates with PREP and circumcision are presented in **Table 2**. Age, education, income, religion, HIV or HSV-2 status were not associated with commitment to PREP or circumcision. However, men who reported higher risk sex or a prior STD were more likely to endorse using PREP but not circumcision.

**Table 2:** Sociodemographic and risk characteristics of participants towards initial commitment to PREP (n = 85) and circumcision (n = 72).^a^

Variable		PREP Commitment		Circumcision Commitment	
	N (%)	N (%)	RR (95% CI)	N (%)	RR (95% CI)
Age					
<30	49 (55.1)	20 (41.7)	ref	3 (6.7)	ref
≥30	40 (44.9)	8 (22.2)	0.53 (0.26–1.08)^d^	5 (19.2)	2.88 (0.74–11.21)
Education					
<Senior Secondary (grade 11)	51 (57.3)	16 (32.7)	ref	3 (7.1)	ref
≥Senior Secondary (grade 11)	38 (42.7)	11 (31.4)	0.96 (0.51–1.82)	5 (17.2)	2.41 (0.62–9.41)
Monthly Income (INR)					
<4500	42 (47.2)	13 (31.7)	ref	3 (8.3)	ref
≥4500	47 (52.8)	15 (34.1)	1.08 (0.58–1.98)	5 (13.9)	1.67 (0.43–6.52)
Religion					
Other than Hindu	16 (17.8)	1 (6.7)	ref	0 (0.0)	
Hindu	74 (82.2)	27 (38.6)	5.79 (0.84–39.77)^d^	8 (11.3)	e
High risk sex^b^					
No	67 (74.4)	17 (26.2)	ref	7 (13.0)	ref
Yes	23 (25.6)	11 (55.0)	2.10 (1.19–3.73)^f^	1 (5.6)	0.43 (0.06–3.30)
Previous STI					
No	77 (86.5)	22 (29.3)	Ref	8 (12.5)	
Yes	12 (13.5)	6 (60.0)	2.05 (1.10–3.80)^f^	0 (0.0)	e
HIV/HSV-2^c^					
No	83 (92.2)	26 (33.3)	ref	7 (10.5)	ref
Yes	7 (7.8)	2 (28.6)	0.86 (0.25–2.90)	1 (20.0)	1.91 (0.29–12.81)

a For PREP analysis, 5 participants were excluded because of missing data and for circumcision commitment analysis 4 participants were excluded because of missing data and 14 participants because of previous circumcision history.

b History of concurrent sexual relationship or contact with female sex worker.

c HIV or HSV-2 seroprevalence.

d p<0.10.

e Model did not converge.

f p<0.05.

#### Willingness to Pay Application

**Figure 2:**
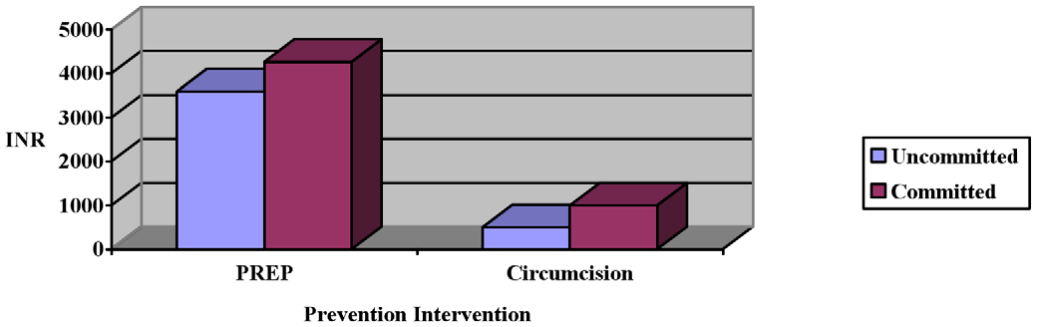
Willingness to pay for PREP and circumcision amongst those with and without initial commitment.^a^ ^ a^Difference between committed and uncommitted willingness to pay for PREP and circumcision did not reach statistical significance by Wilcoxan Rank sum test.

The PREP and circumcision willingness to pay values were right-skewed. The median values for lifetime PREP and circumcision overall were 4071 INR (∼$100) and 550 INR (∼$14) respectively. There was no relationship between personal income and willingness to pay as demonstrated by linear regression models for circumcision (p = 0.99) or PREP (p = 0.58) respectively. The willingness to pay for circumcision and PREP did not differ between participants who demonstrated initial commitment and provided a value for themselves and those who were uncommitted and provided a value for a peer (**Figure 2**). Willingness to pay values amongst participants who were uncommitted to PREP were stratified by sociodemographic and risk characteristics and demonstrated only borderline significant positive associations for younger age (p = 0.06), history of previous STI (p = 0.10) and HIV/HSV-2 status (p = 0.06). Willingness to pay values amongst participants uncommitted to circumcision were also examined and demonstrated a borderline significant positive association with non-Hindus (p = 0.06).

## Discussion

Converging stories reflected in these analyses show that overall initial commitment to circumcision and PREP is low in a general Indian truck-driver population. However, there are several notable psychosocial leverage points within the ARRM framework that may increase commitment to PREP, and less so to circumcision, in high-risk men. The relationship with previous higher risk sex experiences and initial commitment to PREP, and an appeal to family's susceptibility through fear of HIV, are examples of these potential leverage points that might affect later stages in the ARRM. In the labeling phase, perception of risk, and fear offered emerging themes that may yield openings for health care providers and educators. Additionally, initial commitment to circumcision was largely comparable across different strata and risk behavior, whereas there was heterogeneity in initial commitment to PREP across different sub-populations of truck-drivers. Increased commitment to PREP was evident in drivers with previous sexual risk behavior, history of STI and potentially non-muslim religion, younger age and positive HIV/HSV-2 status.

Social norm effects were most evident as direct and indirect norms in the context of initial commitment to circumcision. “Indirect” normative effects are those that reflect what a peer or family member may think about the behavior [48] or in this case about circumcision as an HIV prevention intervention. The proportion of men with initial commitment to circumcision was 11%, which is lower than in studies conducted in Africa where the median acceptability averaged 65% (range 29–87%) [49]. In this study indirect norms were represented largely by the prescriptive beliefs held by peers and family members that exerted influence upon ego and were weighted by the motivation to comply. For example circumcision was largely viewed as a marker of religious identity and proof of belonging to a religion, exemplified by responses to questions about what a family elder thinks of circumcision or how much a peer may be willing to pay for circumcision. The significance of this is compounded by stories of men being identified as Muslim or Hindu during communal strife according to whether or not they were circumcised [50]. In the present analysis this was evident especially amongst uncircumcised men who described stories of killing or burying according to circumcised status. This potential negative response due to religious connotation poses a major barrier to circumcision for HIV prevention in this setting. In contrast to other studies of initial commitment to circumcision [49], there was little focus on pain involved with the procedure, recognition that there would be unchanged sexual pleasure and there was a view of improved hygiene amongst uncircumcised men. These frameworks would likely engender positive associations with circumcision, however, they were all overshadowed by the strong communal norms. In this setting, however, circumcision was seen as less feared as a technique given the high percentage of respondents who were aware of it and who knew another peer who was circumcised. This is in contrast to other settings where circumcision is being introduced to populations that are more homogenous and where circumcision is often less known [51], [52].

Further consideration of the potential influence of patterned inequality of access to things across strata in the present study should be considered, such as in the case of a trend towards increased willingness to pay for PREP with younger ages. For example, others have found that norms generally had stronger influences among younger individuals and among people who have greater access to informational social support, including males, ethnic majorities and people with higher levels of education [53], [54]. Thus efforts to promote circumcision may have to focus primarily on cultural issues rather than benefits or risks of the procedure, and acknowledging the communalism that may persist even after intensive culturally competent educational interventions.

Because truck-drivers have substantial opportunities for social interaction and peer norms are widely shared irrespective of risk behaviors, this study focused on a general population of truck-drivers irrespective of previous risk behavior. A notable finding was that drivers with increased risk were more likely to describe increased initial commitment to PREP. However, there were no differences in willingness to pay for PREP for self or peer, between those with and without initial commitment to PREP. This suggests that there may be similar levels of acceptability amongst peers regardless of whether the specific prevention intervention is designed for themselves or for another driver or peer in need. One driver mentioned “*It would be better if we know about this medicine. Even if it is not useful to us, we can suggest this medicine to our friends.*” These attitudes suggest that to enhance commitment, interventions to affect peer norms and leaders may be required. These “indirect” normative forces have been found to be the summary of the products of beliefs about whether specific people such as peers support a prevention intervention and the individual participant's motivation to comply with those people [54].

Another group who clearly were not at risk for HIV infection or other STIs, but expressed interest in circumcision or PREP was evident from this study. Cautious handling and further education may be required for these individuals for which these interventions would be inappropriate. HIV was the most reported health condition feared and its potential consequences on family may lead individuals at low risk to seek unnecessary prevention interventions which may alter cost-benefit equilibriums of these interventions.

Response and enjoyment efficacy of PREP amongst study participants were conditional on anticipated side effects and benefits as seen in previous studies [22]. Decreased sexual enjoyment through potential PREP side-effects were a prominent finding from qualitative interviews and a major consideration that would affect initial commitment. This concern was also recently found in a different population of men at risk for HIV infection in New England, whereby having no perceived side effects of PREP was one of only four factors that were predictive of intent-to-use [22]. An additional finding from this study which was different from the New England study was the idea of the influence a physician might have upon initial commitment. Whereas in the New England study, recognition of peer's influence on PREP was described, in the current study PREP would be taken if suggested by a physician. This highlights the often prescriptive relationships that patients have with physicians in India [55], whereby physicians are required to navigate complicated social and legal realities in the provision of HIV care [56]. Pills and medications can be powerful in this context [57], [58] and can often be seen as a marker of better care by patients [59]. Therefore, a physician's involvement, or the very nature of pills as HIV prevention, could yield increased initial commitment in the Indian context via response efficacy as compared to non-pill forms of HIV prevention. If PREP is found to be efficacious, increased effectiveness and utilization will require programs to educate physicians about appropriate PREP use. Additionally, HIV prevention in pill form can be secretive and non-stigmatizing a common argument utilized by microbicide advocates [60]. However, considerable challenges would remain requiring additional investigation such as the concept of taking pills chronically which often clashes with the symptomatic disease model in India where patients often decide that they do not need medications when they feel well [61].

Cost was a major barrier to initial commitment of both prevention interventions examined in this study. Pilot testing for this study demonstrated that existing local costs had to be decreased by 30 fold for PREP and 3 fold for circumcision in order for the respondents to be able to choose a cost category that they would be willing to pay. Cost has been found to be a major barrier to acceptability of circumcision in Sub-Saharan Africa [49]. In one circumcision acceptability study in Kenya, 34% of participants who initially stated that their preference was to remain uncircumcised changed their minds when the proposed cost of the procedure was set at US$3.00 [62]. In a cohort of men who have sex with men, cost was a significant predictor of intent-to-use PREP with not having to pay for PREP as one of four statistically significant predictors of this initial commitment [22]. Both value ranges that were utilized in this study are far below actual cost in local settings and suggest that these prevention interventions will require supplementation by public health stakeholders. In our models of cost of PREP, we utilized a very conservative figure based upon only 50% adherence and termination of PREP at the age of 50. In one PREP cost-effectiveness study a high HIV incidence rate coupled with increased PREP efficacy to 90% would likely yield larger reductions in lifetime infection risk [63], and suggests that if PREP were to be used on a daily basis as in this study, subsets of populations with high-risk sexual risk behaviors would benefit most.

Low self-efficacy reported by men in this occupation represents a major obstacle to the support and commitment towards novel HIV prevention interventions such as circumcision and PREP. Drivers uniformly reported marked challenges by the work schedule, stressors at work and time away from home and little ability to guide one's decisions. Poignant descriptions of decreased autonomy in decision making coupled with the increased economic pressure of the profession and the role this has on lower self-worth and poor societal perceptions of the profession (ie. inability to get married) have been described recently for truck-drivers [64]. Such factors make self-efficacy one of the initial commitment sub-domains that seems least likely to be leveraged. More robust and multi-level interventions may be required such as structural interventions that provide adequate and hygienic rest stops or a mechanism to bargain with truck owners and other authorities.

Our study had limitations. First we were examining initial commitment to two HIV prevention interventions and not the third enactment stage of the ARRM. A major assumption of the model rests on the premise that proper “labeling” and “developing commitment” will ultimately lead to a reduction of risk behavior, which has been demonstrated on multiple occasions in multiple settings [65], [66], [67]. Because the interventions examined in this study – circumcision and PREP - are either under investigation or currently unavailable for HIV prevention in India, actual uptake of the interventions could not be assessed. We did not directly ask respondents about behavioral disinhibition and risk-compensation, however, these themes did not emerge on their own, and will be needed to be addressed in further studies of HIV prevention interventions that are found to be modestly effective. Finally, our participation rate at 44.1% is low. However, this rate reflects a similar participation rate (50%) in this population from our previous government truck-driver HIV prevention program *Surakshitham* (unpublished data) which included behavioral change communication and did not include HIV testing.

Despite these limitations, our findings suggest that the ARRM may be useful in identifying several leverage points that may be used by peers, health care providers and public health field workers to enhance initial commitment to novel HIV prevention interventions in India. The study utilized a theory driven approach and is the first to examine initial commitment towards two novel HIV prevention interventions within the same population, allowing an ability to uncover paradox and contradiction. The use of complementary approaches to initial commitment including qualitative, quantitative and a willingness to pay application offered the ability to measure overlapping but also different facets of this phenomena, yielding an enriched, elaborated understanding of initial commitment to PREP and circumcision.

## Supporting Information

Figure S1 (0.05 MB DOC)
