# Supplementary material for: Initial Commitment to Pre-Exposure Prophylaxis and Circumcision for HIV Prevention amongst Indian Truck Drivers
Source: PLoS One. 2010 Jul 30;5(7):e11922. doi: 10.1371/journal.pone.0011922 (PMC2912853; doi:10.1371/journal.pone.0011922)
Supplement: Figure S1 — (0.05 MB DOC) [file pone.0011922.s001.doc]

**APPENDIX 1**

*Health, Self-Efficacy and Fear*

What does it mean to you to be healthy?

Probe: [What does a healthy person look like? What are the qualities of a healthy person?]

What does it mean to you to be sick?

Probe: [How does a sick person look?]

What do you think makes people sick?

How do you think people can prevent getting sick?

Probe: [Where did you hear that?]

What health conditions or diseases do you fear?

Probe: [Why?]

Probe: [What would happen if you got this disease?]

Are there things that you do right now to take care of your health?

Probe: [What do you do?]

Are there things that you think you should be doing to take care of your health, but you aren’t?

Probe: [What are these things?]

[What prevents you from doing these things?]

How has being a truck driver affected your overall health?

After becoming a truck driver has your ability of taking care of your health changed?

Probe: [Tell me about it.]

What do you think are the major health issues/ diseases affecting truck drivers?

Probe: [Why do you think they had this problem?]

[Why do you think these are the major health problems?]

[Has anyone talked to you about this?]

How do you think stress/tension/worries affect your health?

Probe: [Do you have a lot of stress/tensions/worries that affects your health?]

[Tell me about it. What are the stresses? (cut them if they go on and on)] [What kind of stresses?]

[Do you think that stress can cause HIV?]

[If they know someone with HIV ask them whether they think that stress caused HIV in their case?]

*Knowledge of HIV and STDs, Perception of Risk and Social Norms*

What does the term HIV/AIDS mean to you?

Probe: [How did you hear about it?]

[What have you heard?]

[Has anyone talked with you about it?]

Do you think HIV is affecting truck drivers?

Probe: [In your company? In other companies?]

Have you ever heard about STD or sexual diseases?

Probe: [Tell me more about what you think these are]

[Have you heard that these type of diseases come from having various relationships?]

[What kind of sex would lead to what kind of illnesses]

Do you know if these problems can be treated?

(Here give them facts after they respond)

Have you ever experienced psychological or physical pain from an STD before?

Have you ever been to a clinic to seek treatment for these type of diseases?

Probe: [Or has a doctor prescribed tablets or injections?]

[How long ago was the last time that this happened?]

Due to unavoidable circumstances or being separated from family one may have committed a mistake once or twice that can happen to anyone. Do you think that this could happen to you?

Probe: [If this has happened, kindly tell me about it.]

Probe: [Have you ever had sexual contact other than your wife(or lifetime partner)?]

Do you have any driver friends who have acquired HIV or STDs?

Probe: [How close were they to you?]

Probe: [How typical is this amongst other truck drivers you know]

Is there any possibility that you may have ever had sex with a close male friend.

Probe: [Or trucker helper/cleaner?]

Any sexual contact with old girlfriend like from your village.

Probe: [Do you have any occasional sexual partner or partner who is known to you.]

Do you feel like you are at risk for HIV?

Probe: [Why, or why not? Please tell me more about it.]

In the past 6 months or one year what type of sexual experiences have you had with your life partner?

Probe: [Are you at risk from this relationship?]

In your previous contacts with your sexual partners, what are the prevention processes that you have maintained for STDs or HIV?

Probe: [Some people think that washing the genitals after sex can prevent HIV/STIs, do you believe this? Do you practice this?]

[Some use nirodh to prevent HIV/STI, do you believe or practice this?]

[What about choosing sex partners who you think have less risk for HIV. What types of people might this be?]

[What about trusted friends telling you about sex partners? Is this helpful in preventing HIV?]

Do you currently maintain prevention processes for HIV/AIDS and STDs?

Probe: [If so, which ones]

Probe: [Are you able to prevent HIV? Why or why not?]

Do you have sexual contact with women who have fewer sexual relationships?

Probe: [In this circumstance have you ever used a condom?, why or why not?]

[Do you prefer sexual relationship with a woman who has never had sex with others?]

Like a virgin, a neighborhood girl, first time sex worker.

[Do you think that this woman is less likely to have disease? Why or why not?]

*Circumcision Response and Enjoyment Efficacy*

Have you undergone circumcision during your childhood or as an adult?

Probe: [If so, when and why?]

Do you know why the foreskin on the penis is cut?

What do the elder members in your family think of circumcision?

What do your friends think of circumcision?

Do you have any known person who is circumcised?

Generally, in what age do people prefer to go for circumcision?

Generally what type of people prefer to go for circumcision?

Probe: [Is there any community or religion base]

*{{Some studies in different parts of the world have found that circumcision can help to reduce the risk of HIV by half. Circumcision is a minor surgical procedure and can help reduce the risk of men getting HIV, however a condom would still be required. Also, if the man is already HIV positive, he will still have to use condom while having sex to prevent other people from getting HIV. Circumcision may not prevent HIV transmission to wife if man is positive and may not prevent some other STD transmission. Do you have any questions about this?

[Focus on reducing risk by half, one time procedure, may not prevent HIV transmission to wife if man is positive, man will still need to wear a condom, and may not prevent other STDs]}}

What do you think about this? What other questions do you have?

Are you ready to get circumcised to reduce your risk for getting HIV?

Do you think that circumcision is good for health? Why or why not?

Why do you think circumcision might decrease the chance that you will get an STD or HIV?

Probe: [What does the foreskin have or do that makes one more likely to get HIV]

Do you feel that circumcision would/does affect your health?

Probe: [Would it provide any health problems? If so which ones?]

Would/does circumcision affect ability to have sex?

Probe: [Any chance for sexual dysfunction because of circumcision?]

[Does circumcision make sex more pleasurable?]

Does circumcision make the penis more or less clean?

Probe: [Why?]

What sorts of people might be interested in circumcision to prevent HIV?

We have been talking about circumcision. Tell me in your own words what circumcision is. How is it done? Why would people have it done? And what are your fears if any about circumcision.

*PREP Response and Enjoyment Efficacy*

Have you used any tablets before or after having sex to prevent HIV?

Probe: If so, which ones?

Before using any tablets, have you consulted an RMP, or have you taken advice from the medical shop.

**{{Some medications may be available in tablet form that can be taken once daily to decrease the risk of HIV infection by half. This is called PREP. The medication would have to be taken once a day while you are sexually active to have an effect. If they are stopped they may not protect the person from getting HIV. Condoms would still have to be used if this pill were to be taken.}

What do you think about this? Do you have any questions about PREP?

Are you ready to use PREP to reduce your risk for getting HIV?

Tell me about why you would use it?

Probe: [What other sorts of people might use this medication]

Why might other people use this medication?

What concerns would you have about using this medication?

We have been talking about PREP to prevent HIV by 50%. Tell me in your own words how this works? Why would people take this medicine? How do they take it? How often? What are your fears about taking this medicine? What other questions would you have before taking this medicine?
